# *The Organon* and Materia Medica Club of the Bay Cities of California

**Published:** 1897-02

**Authors:** W. E. Ledyard

**Affiliations:** Secretary


W. E. Ledyard, B. A., M. B., etc.,
*Secretary.*

Reported by Eleanor F. Martin, M. D.

---

### *THE ORGANON* AND MATERIA MEDICA CLUB OF THE BAY CITIES OF CALIFORNIA.

The regular semi-monthly meeting was held at the office of Dr. George H. Martin, 606 Sutter Street, San Francisco, Friday evening, August 21st, 1896.

Members present: Drs. J. M. Selfridge, W. E. Ledyard, A. McNeil, C. M. Selfridge, M. T. Wilson, M. F. Underwood, George H. Martin. Visitors: Drs. C. V. C. Scott and Caroline L. Guild.

The meeting was called to order at 8.15 by the President, Dr. J. M. Selfridge. The minutes of the previous meeting were read by the Secretary, and after corrections by Drs. J. M. Selfridge and McNeil, were approved.

The question of admitting women as members was postponed until the next meeting.

Dr. J. M. Selfridge commenced the reading of *The Organon* at Section 182 continued to 185.

### Discussion.

Dr. Ledyard—I should like to ask if any of the physicians here have had any experience in antidoting by potentizing the crude drug that was used internally or locally, or both internally and locally, and giving it internally?

Dr. Martin—I have had a good deal of experience in syphilitic cases where Iodide of Potassium and Mercury were used. When large doses of these drugs were used I have given them in small does internally, and have had good results.

Section 186.

### Discussion.

Dr. Ledyard—In regard to local treatment in eye troubles. Several cases I have lately seen, seem to impress me with the fact, that in one case, cataract in both eyes resulted from local treatment. In two cases, opacity of the cornea was apparently produced in the same way. One case of granular lids which were cauterized by an oculist, has had opacity of the cornea every since. I know of another case in which, two weeks after an injury to the right eye, the eye-ball was enucleated and an opacity subsequently appeared on the left cornea after local treatment.

Dr. J. M. Selfridge—I have not a doubt but that local applications to the eyes are injurious. I have known Nitrate of Silver used, strong, to remove granulations of the lids. I have also had cases in which no treatment had been used at all, and yet there were opacities of the cornea.

Dr. Ledyard—I mean to suggest that this may be one of the causes of opacity of the cornea. There are medicines which produce this condition, such as Argentum-nitricum, Calcarea-ostrearum, Atropine, etc.

Dr. C. M. Selfrldge—Would you not give Arnica for injury to the eyes?

Dr. Ledyard—Yes, if the symptoms called for it.

Dr. Wilson—Would you use the remedy locally?

Dr. Ledyard—It would not be necessary to use it locally.

Dr. C. M. Selfridge—I met an old Indian who had been struck in the eye by the tail of a fish, resulting in opacity of the cornea. He had Arnica symptoms and I gave him six or eight powders of Arnica$^{200}$, three a day. His sight returned in a week.

Dr. McNeil—I had an epileptic, thirty years of age. At twenty years of age he had, what he termed, "sore eyes." The eyes got better after considerable local treatment. He then had an epileptic fit every four weeks, then closer and closer together, until he had them several times a day. Nature or the vital force had manifested the trouble at the eyes. This manifestation was suppressed by the local treatment and went to the brain. Such cases are not very rare.

Dr. Martin—I knew of a family of three children with eczema capitis. Two of the children were treated locally by allopathic physicians, and they both died in convulsions. I treated the third one. It was the worst case of eczema capitis I have ever seen. The little one is now a healthy child. I believe the first two children died from the "skin phase" of the disease being suppressed.

Dr. J. M. Selfridge—I had a similar case in which tar ointment was used. The child became drowsy and showed symptoms which frightened the mother. She washed the ointment off and brought the child to me and I cured it.

The meeting was then declared adjourned until the first Friday in September, when the meeting would be held at Dr. Martin's office, commencing *The Organon* at Section 187.

